# Acute Myocardial Ischemia Secondary to Embolization of Left Atrial Myxoma to Coronary Artery

**DOI:** 10.7759/cureus.9260

**Published:** 2020-07-18

**Authors:** Mohammed Al-Musawi, David Rubay, Levonti Ohanisian, Slee Yi, Suhad AlOmaishi

**Affiliations:** 1 Surgery, Anschutz Medical Campus, University of Colorado, Aurora, USA; 2 Surgery, Charles E. Schmidt College of Medicine, Florida Atlantic University, Boca Raton, USA; 3 Orthopaedic Surgery, Morsani College of Medicine, University of South Florida, Tampa, USA; 4 Internal Medicine, Life Alliance Organ Recovery Agency, University of Miami, Miami, USA

**Keywords:** myxoma, st elevation, myocardial infarction, dca

## Abstract

Cardiac myxoma is the most common primary cardiac tumor. Rarely it can present with systemic or coronary embolization due to fragmentation of the tumor mass. We present a case of a young male who presented with an acute myocardial ischemia secondary to embolization of a left atrial myxoma originating from the left atrium. The patient underwent successful emergency surgical management of both the myxoma and the occlusion of the coronary artery. In this scenario, the surgery is the only effective treatment. The case also highlights the significance of performing emergency echocardiography in the setting of acute myocardial ischemia to look for possible associated pathology which can inform management plan.

## Introduction

Acute myocardial infarction (AMI) is caused by non-atherosclerotic lesions in 1-12% subset of cases [1]. One such cause includes embolization [1]. Sources of embolization include a mural thrombus, a septic embolus secondary to infective endocarditis, valve calcification or from a left atrial myxoma [2-4]. Although rare, embolization in the coronary artery is a possibility which cardiologists and cardiac surgeons should be aware of [5-7]. In one case series that spanned over 10 years they reported five cases of coronary embolization, including one case secondary to a left atrial myxoma embolus [1]. In cases of an embolus secondary to a thrombus, patients often undergo a combination of interventions including systemic anticoagulation, coronary wiring and conventional aspiration thrombectomy devices [8-10]. Cases of acute coronary syndrome secondary to septic embolus from intracardiac infected valves have been managed with combined aspiration thrombectomy and coronary stenting [11, 12]. Having intracardiac pathology as the source of the embolization such as a left atrial myxoma or infected left-sided valve is a valid justification for surgical intervention of direct surgical thrombectomy with valve replacement [2, 13]. A review of the literature demonstrates no guideline or standard management for coronary artery thromboembolism. We intend to add to the literature with our case presentation and treatment method in order to increase the cases available in the literature and hopefully improve standard management of similar cases in the future [2, 14].

## Case presentation

**Figure 1:**
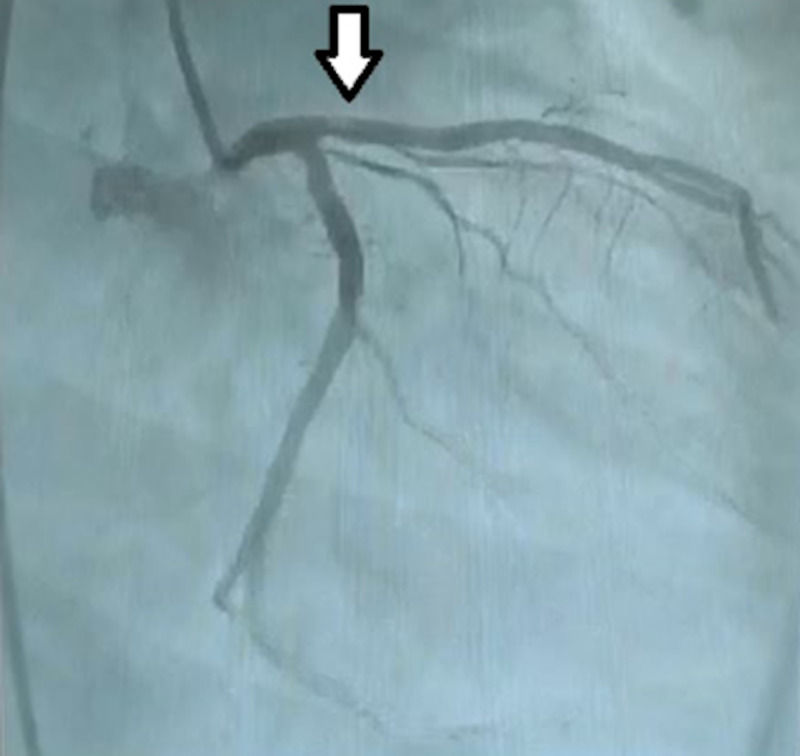
Filling defect in LAD LAD: Left anterior descending artery

**Figure 2:**
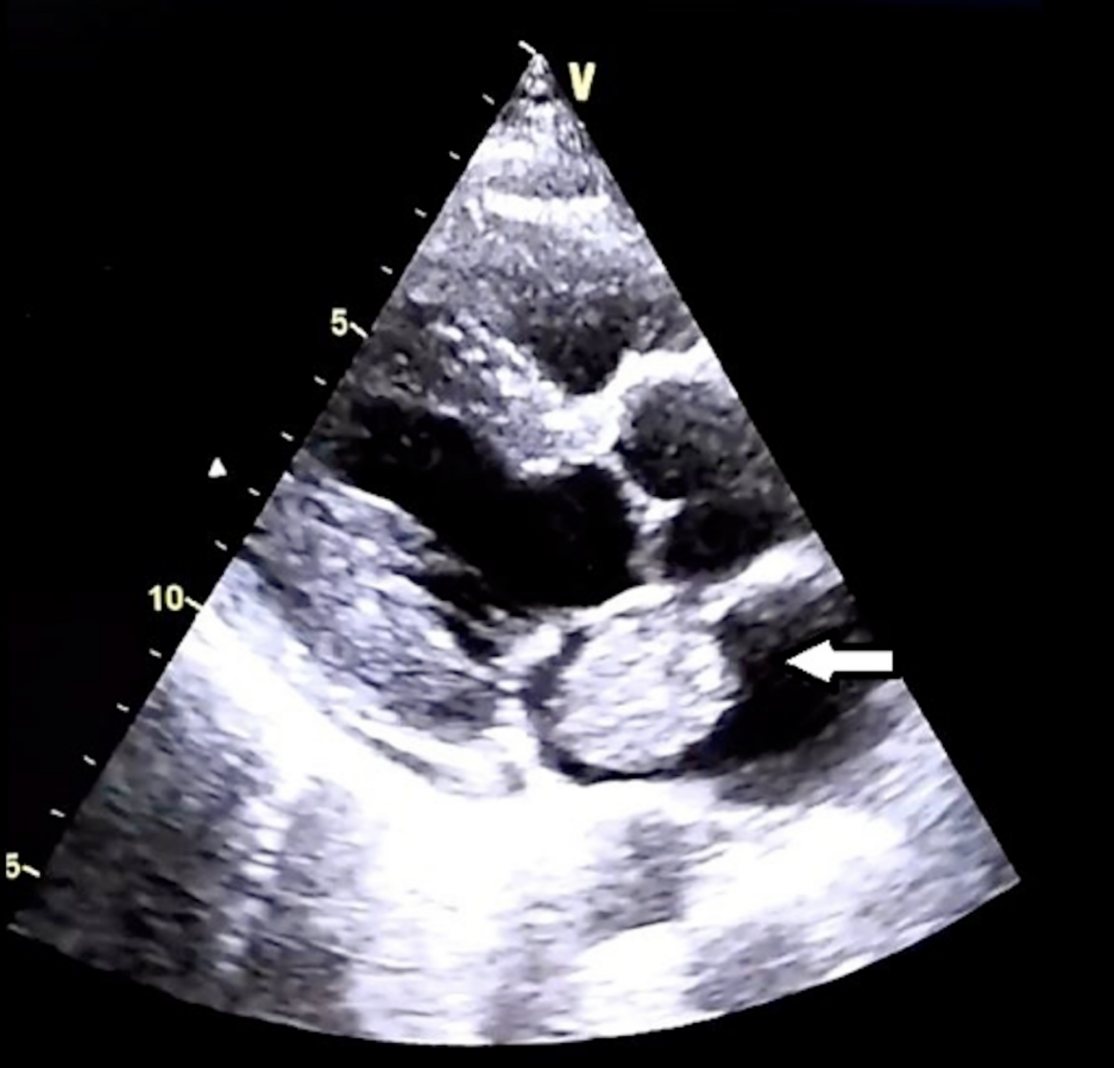
Mobile mass in left atrium

The patient is a 25-year-old male who was referred from cardiology with a diagnosis of acute ST-elevation myocardial infarction (STEMI), altered consciousness and acute renal impairment. From the onset of symptoms, time to angiography was two hours. Angiography demonstrated a left anterior descending artery (LAD) filling defect (Figure 1), and an echocardiogram performed showed a mobile left atrial mass (Figure 2) attached to the interatrial septum.

ECG showed ST elevation in the anterior chest leads with a positive troponin. Due to the multisystem nature of presentation, along with evidence of a mobile mass in the left atrium, it was suspected that the mass was the primary lesion responsible for showering small pieces throughout the vascular tree. The patient was taken to the operating room with plans to remove the mass from the left atrium and place a graft to the LAD to overcome acute ischemia and avoid MI. Using aortocaval cannulation and cardioplegic arrest on aortic cross clamp and moderate hypothermia, the left mammary artery (LIMA) was harvested. The left atrium was opened and a large 3 cm x 3.5 cm myxoma was excised and the left ventricle irrigated with normal saline to remove any missed detached particle. The LIMA was anastomosed to the LAD and routine rewarming and deairing of aortic root was completed. The heart began to beat spontaneously after removing the cross clamp. The patient was admitted to the cardiac intensive care unit (CICU) after surgery for 48 hours. Consciousness and renal function began recovering gradually. The mass was sent for histopathologic examination and returned with the diagnosis of myxoma. The patient spent another seven days in the ward and was discharged home in good health.

## Discussion

Acute myocardial ischemia can be caused by non-atherosclerotic lesions in 1-12% subset of cases, one of those rare causes is embolization [1]. Sources of embolization include a mural thrombus, a septic embolus secondary to infective endocarditis, valve calcification or from a left atrial myxoma [2-4]. Although rare, embolization in the coronary artery is a possibility which cardiologists and cardiac surgeons should be aware of [5-7]. In one case series that spanned over 10 years they reported five cases of coronary embolization, including one case secondary to a left atrial myxoma embolus [1]. In cases of an embolus secondary to a thrombus, patients can undergo a combination of interventions including systemic anti-coagulation, coronary wiring and conventional aspiration thrombectomy devices [8-10]. Cases of acute coronary syndrome secondary to septic embolus from intracardiac infected valves have been managed with combined aspiration thrombectomy and coronary stenting [11, 12]. Having intracardiac pathology as the source of the embolization such as a left atrial myxoma or infected left-sided valve is a valid justification for surgical intervention of direct surgical thrombectomy with valve replacement [2, 13]. A review of the literature demonstrates no guideline or standard management for coronary artery thromboembolism. We intend to add to the literature with our case presentation and treatment method in order to increase the cases available in the literature and hopefully improve standard management of similar cases in the future [2, 14].

## Conclusions

Acute embolic STEMI is a reported complication of left atrial myxoma. Surgical removal of the mass can successfully control the source of embolic occlusion of coronary arteries and save myocardium.
